# Novel Therapeutics for Malaria

**DOI:** 10.3390/pharmaceutics15071800

**Published:** 2023-06-23

**Authors:** Haitham Alaithan, Nirbhay Kumar, Mohammad Z. Islam, Angelike P. Liappis, Victor E. Nava

**Affiliations:** 1 Veterans Affairs Medical Center, Washington, DC 20422, USA; angelike.liappis@va.gov; 2 Department of Medicine, George Washington University, Washington, DC 20037, USA; 3 Department of Global Health, Milken Institute of Public Health, George Washington University, Washington, DC 20037, USA; nkumar@email.gwu.edu; 4 Department of Pathology and Translational Pathology, Louisiana State University Health Science Center, Shreveport, LA 71103, USA; mohammad.islam@lsuhs.edu; 5 Department of Pathology, George Washington University, Washington, DC 20037, USA

**Keywords:** malaria, *Plasmodium*, novel, antimalarial drug

## Abstract

Malaria is a potentially fatal disease caused by protozoan parasites of the genus *Plasmodium.* It is responsible for significant morbidity and mortality in endemic countries of the tropical and subtropical world, particularly in Africa, Southeast Asia, and South America. It is estimated that 247 million malaria cases and 619,000 deaths occurred in 2021 alone. The World Health Organization’s (WHO) global initiative aims to reduce the burden of disease but has been massively challenged by the emergence of parasitic strains resistant to traditional and emerging antimalarial therapy. Therefore, development of new antimalarial drugs with novel mechanisms of action that overcome resistance in a safe and efficacious manner is urgently needed. Based on the evolving understanding of the physiology of *Plasmodium*, identification of potential targets for drug intervention has been made in recent years, resulting in more than 10 unique potential anti-malaria drugs added to the pipeline for clinical development. This review article will focus on current therapies as well as novel targets and therapeutics against malaria.

## 1. Malaria

Malaria is a potentially lethal parasitic disease caused by unicellular protozoa of the genus *Plasmodium*. Among more than 150 *Plasmodium* species that infect vertebrates upon transmission by *Anopheles* mosquitoes, only four (*P. falciparum*, *P. vivax*, *P. ovale* and *P. malariae*) utilize humans as a natural host. However, well-known causes of simian malaria, such as *P. knowlesi* and *P. cynomolgi*, have been isolated from humans and are currently considered zoonotic diseases [1,2]. Despite global initiatives led by the World Health Organization (WHO) to reduce the impact of the disease, it remains endemic in vast areas of the tropics (in 85 countries) and represents 7.8% of the global burden of disease worldwide. Therefore, malaria is the most important parasitic disease globally, producing at least 247 million cases (95% in Africa) and 619,000 deaths in 2021 alone [3].

### 1.1. Parasite Life Cycle and Clinical Manifestations

Malaria is naturally transmitted from human to human by the female *Anopheles* mosquito. Sporozoites enter the human blood stream during a blood meal and migrate to the liver, where they mature to schizonts within hepatocytes. Infected hepatocytes rupture, releasing daughter merozoites that invade red blood cells (RBCs) in the circulation. In the erythrocytic stage, the parasites divide asexually, causing cyclic hemolysis and further release of merozoites. Rupture of RBCs usually coincides with the clinical symptoms of malaria as that leads to anemia and release of merozoites together with proinflammatory molecules. However, some of the merozoites exit this erythrocytic phase and mature inside the RBCs to male and female gametocytes and continue their sexual life cycle in the female *Anopheles* mosquito upon subsequent blood meals. In addition to the replicative phase in the hepatocytes (the exoerythrocytic stage), *P. vivax* and *P. ovale* may remain dormant in the liver as hypnozoites and resume asexual replication several years after the initial infection. Rare methods of malarial transmission have been reported, including blood transfusion, needle sharing, vertical transmission during pregnancy or birth, and organ transplantation [4,5].

Clinical manifestations vary depending on age of the patient, immune status, and parasite species (length of the asexual intraerythrocytic life cycle: 24 h in *P. knowlesi*, 72 h in *P. malariae*, and 48 h in other species). People in high transmission areas usually develop clinical immunity (premunition) because of repeated infections and present with minimal to no symptoms. Non-immune individuals tend to be symptomatic and at increased risk of severe disease. Mild (uncomplicated) malaria presents with non-specific manifestations such as periodic fever, chills, myalgia, headache, vomiting, diarrhea, and anemia. The classical paroxysms of malaria include a succession of chills, rigors, fevers, and profuse sweating as the temperature returns to baseline and correlate with the erythrocytic cycle of the parasite. Severe (complicated) malaria can present with severe anemia, lactic acidosis, organ dysfunction (pulmonary-, renal-, and neurologic-only with *P. falciparum*) and coma (Table 1), which is considered a medical emergency requiring immediate attention and care.

### 1.2. Diagnostic Modalities

The signs and symptoms of malaria are nonspecific and overlap with other febrile and traveler illnesses. In a case series of 95 patients (62 adults and 33 children) returning to the US with malaria, 13% were initially misdiagnosed [7]. Most cases of malaria diagnosed in the US are acquired from Africa (87.8%), followed by Asia (8.7%), South America (1.8%), Central America and the Caribbean (1.5%), and less than 1.0% from Oceania [8]. The WHO recommends parasite-specific laboratory testing when malaria is suspected [6]. Commonly used laboratory tests include microscopic examination of stained blood smear, rapid parasite antigen detection tests (RDTs), and nucleic acid amplification test (NAAT) [9]. Thick and thin smear microscopy remains the gold standard for malaria diagnosis, allowing parasite quantification and identification to the species level at a low cost when adequately trained laboratory personnel are available. It is recommended that repeat blood films be obtained and examined every 6 to 8 h for up to 3 days upon clinical suspicion [10,11,12].

## 2. Antimalarial Drugs

**Figure 1:**
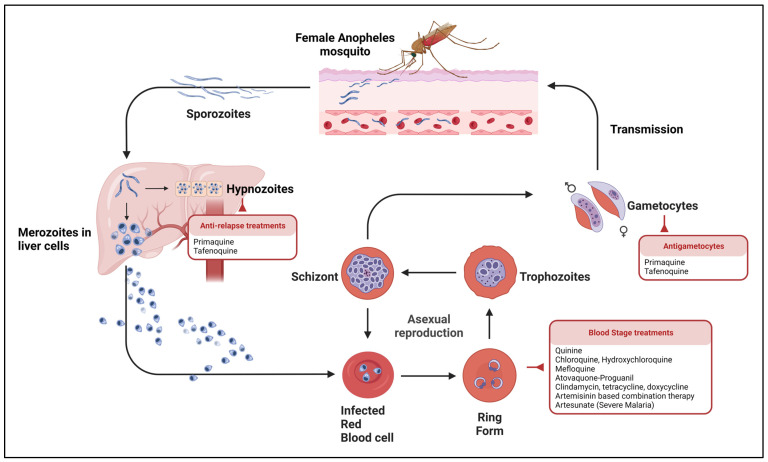
Malaria life cycle and current therapies. Created with BioRender.com.

Available antimalarial drugs are many, including quinolones, antifolates, artemisinin derivatives, naphthoquinones, trioxolanes, and antimicrobials such as lincosamide, tetracycline, and macrolides (Table 2). Unfortunately, a single antimalarial agent effective against all species and all sexual and asexual stages of *Plasmodium* is yet to be developed. Most antimalarial drugs exhibit considerable stage and species selectivity (Figure 1), mandating combined anti-malarial regimens for proper efficacy and to prevent the development of resistance.

### 2.1. Current Treatments

Periodic fever has been recognized since antiquity and was empirically treated by the Quechua of Peru with ground bark of *Cinchona* trees, from which quinine was extracted in 1820. Formal therapeutic testing started only in the late 19th century after Charles Laveran discovered *Plasmodium* as the cause of malaria. Quinolines became the standard therapy for malaria between the 1920s and 1940s [13]. Although the exact mechanism of action of these drugs is unclear, interference with hemoglobin metabolism leading to toxic accumulation of heme and the inhibition of glycolytic enzymes have been proposed to explain anti-malarial activity in infected erythrocytes [14]. Quinine was the first widely used alkaloid to treat malaria; however, its significant toxicity and the emergence of *P. falciparum* resistance limited its use, promoting introduction of other quinolines including primaquine, mepacrine, and chloroquine.

Chloroquine was developed during World War II and became the anti-malarial drug of choice due to its safety, cost, and efficacy. Development of resistance by *P. falciparum*, first reported in 1957 [15] and later spreading throughout the world, has limited its use to selected areas. Primaquine phosphate and recently approved single-dose tafenoquine (8-aminoquinolines) are medications with activity against *P. vivax* and *P. ovale* hypnozoites and prevent relapse. 8-aminoquinolines must be used with caution in people with glucose-6-dehydrogenase enzyme (G6PD) deficiency due to increased risk of hemolytic anemia. Pyronaridine is another quinoline derivative used in combination with artesunate with good efficacy against uncomplicated *P. falciparum* malaria [16]. Atovaquone, a naphthoquinone, is the first approved anti-malarial which targets *Plasmodium* mitochondria by inhibiting cytochrome B of the bc1 complex and prevents transmission from mosquitos to humans by affecting the sexual stage of the parasite [17]. Usually, atovaquone is used in combination with other drugs, such as proguanil, and has shown effectivity both for curative treatment and chemoprophylaxis [18,19].

Antifolates are the second class of medications to combat malaria and are considered shizonticidals. There are three commonly used drugs in this group: sulfadoxine, pyrimethamine, and proguanil. They are usually combined with other anti-malarial drugs such as artesunate + sulfadoxine–pyrimethamine for treatment or atovaquone + proguanil for chemoprevention [6]. They act mainly by inhibiting enzymes necessary to produce active folates, which in turn serve as essential co-factors in nucleic acid biosynthesis and cell division [20]. Sulfadoxine inhibits dihydropteroate synthase, while pyrimethamine and proguanil inhibit dihydrofolate reductase.

Artemisinin, a sesquiterpene lactone endoperoxide, and its derivatives including artemether, dihydroartemisinin, arteether, and artesunate are first-line therapies for uncomplicated and complicated (severe) malaria. Artemisinin was extracted in 1972 from *Artemisia annua.* Full understanding of its mechanism of action is still evolving, but it involves interaction with the heme group inside RBCs, generating free radicals that mediate protein alkylation and DNA damage [21], resulting in inhibition of parasitic molecules (calcium ATPase and proteosomes) [22]. A uniquely attractive feature of artemisinin lies in its wide spectrum towards all intraerythrocytic phases of *Plasmodium*, including gametocytes, which results in transmission blockade [23]. In addition, artemisinin has shown effectivity against chloroquine- and mefloquine-resistant strains [24,25]. However, downsides of artemisinin and its derivatives include their short half-life, poor bioavailability, lack of activity against latent phases of the parasite (hypnozoites), and emergence of resistance especially in parts of Southeast Asia and Africa. Therefore, it is recommended that artemisinin derivatives be combined with other antimalarial agents to prevent treatment failure and recrudescence [6]. The WHO recommends the following artemisinin-based combination therapies (ACTs): artemether–lumefantrine, artesunate–amodiaquine, artesunate–mefloquine, artesunate–pyronaridine, artesunate–sulfadoxine–pyrimethamine, and dihydroartemisinin–piperaquine. Artesunate, which is a semisynthetic derivative, is accepted as a first-line agent for severe malaria and can be used as monotherapy but must be followed by ACT.

**Table 2:** Current antimalarial drugs.

Class	Category	Drugs	Side Effects	Mechanism of Action	Ref.
Quinoline	*Cinchona* alkaloid	Quinine, quinidine	Hypoglycemia, cinchonism (tinnitus, hearing loss, visual disturbances, headache, dysphoria, nausea, vomiting), hypotension	Toxic accumulation of heme inside the infected red blood cell and inhibition of glycolytic enzymes	[14,26]
	4-AQ	Chloroquine	Pruritis, nausea, vomiting, diarrhea, keratopathy, retinopathy (with prolonged use)		[14,27,28]
		Amodiaquine	Hepatitis, myelotoxicity, agranulocytosis		[29,30]
		Piperaquine	Headache, dizziness, nausea, abdominal pain		[31]
	8-AQ	Primaquine, tafenoquine	Hemolytic anemia in G6PD deficiency	Inhibit pyrimidine synthesis and disrupt mitochondrial electron transport chain by producing oxidative metabolites	[32]
	Quinoline-menthol	Mefloquine	Dizziness, anorexia, vomiting	Toxic accumulation of heme inside the infected red blood cell	[33,34]
	Aryl amino-alcohol	Lumefantrine, halofantrine	Nausea, vomiting, diarrhea, pruritis, rash, cardiac toxicity (with halofantrine)		[35]
	Benzonaphthyridine derivative	Pyronaridine	Headache, vomiting, abdominal pain, bradycardia, hypoglycemia		[36]
Naphthoquinones	Hydroxynaphthoquinone	Atovaquone	Nausea, vomiting, diarrhea, headache, fever, transient liver enzyme elevation	Inhibit the electron transport system at the cytochrome bc1 complex	[17]
Antifolate	Diaminopyrimidine	Pyrimethamine	Bone marrow suppression, glossitis, stomatitis, exfoliative dermatitis, hair loss	Interfere with DNA synthesis by inhibiting DHFR, parasite mitochondrial toxicity seen with proguanil	[20,37]
	Biguanide	Proguanil			
	Sulfonamides	Sulfadoxine	Fever, arthralgia, bone marrow suppression, SJS, hemolytic anemia in G6PD	Interfere with DNA synthesis by inhibiting DHPS enzyme	[38,39]
Artemisinin	Sesquiterpene lactone endoperoxides	Artemether, artesunate, dihydroartemisinin	Nausea, vomiting, diarrhea	Protein alkylation and DNA damage by free radical generation	[21,40]
Trioxolanes	Trioxolane peroxide	Arterolane	Headache, nausea, vomiting	Inhibit heme detoxification and *Pf*ATP6	[41]
Antimicrobials	Lincosamide	Clindamycin	Rash, DRESS, SJS, diarrhea, *C. difficile*	Interfere with protein translation	[42]
	Tetracycline	Doxycycline, tetracycline	Nausea, vomiting, diarrhea, epigastric pain		[43]
	Macrolide	Azithromycin	Nausea, vomiting, diarrhea		[44]

Aminoquinolines (AQ), dihydrofolate reductase (DHFR), dihydropteroate synthetase (DHPS), drug reaction with eosinophilia and systemic symptoms (DRESS), glucose-6-phosphate dehydrogenase (G6PD), *Pf*-encoded sarcoplasmic endoplasmic reticulum calcium ATPase (*Pf*ATP6), and Stevens-Johnson syndrome (SJS).

### 2.2. Plasmodium Drug Resistance

Despite great efforts to develop new antimalarial drugs, resistant *Plasmodium* strains continue to evolve. The parasite’s capacity to genetically mutate to adapt and survive in response to new treatments has led to the emergence of drug resistance, which is defined as the ability of *Plasmodium* to survive or multiply despite the administration and absorption of a drug given in doses equal to or higher than those usually recommended [45]. Phenotypical resistance presents as delayed parasite clearance or treatment failure. According to the WHO, three species have shown drug resistance: *P. falciparum*, *P. vivax*, and *P. malariae* [46], and resistance has emerged to all known anti-malarial drugs. For example, artemisinin-resistant *P. falciparum* is prevalent in the Greater Mekong subregion [47,48] and areas in Africa [49] and has been documented to harbor mutations in the Kelch 13 (K13) gene. K13 encodes Kelch protein, which has a wide array of functions, including facilitating polyuiquination leading to protein degradation in the proteosome [50,51]. Strains with K13 mutations show increased levels of phosphatidylinositol 3-kinase (PI3K) and phosphatidylinositol-3-phosphate (PI3P), which leads to reduced artemisinin sensitivity [51]. Despite resistance, combination therapy including artemisinin retains efficacy so far, largely due to *Plasmodium* sensitivity to partnering drugs.

### 2.3. Novel Therapies

The treatment of malaria is anticipated to become more challenging in the future due to progressive resistance limiting the efficacy of current anti-malarial drugs. More than US $3.0 billion dollars has been spent throughout the world in 2021 alone to combat malaria using mainly drug development and vector control [3]. An optimal antimalarial agent should act on novel targets, lack cross resistance with current anti-malarial drugs, inhibit all stages (asexual, sexual, and latent hepatic stages-of *P. vivax* and *P. ovale*), be effective in a single dose, act rapidly, and be clinically safe [52].

Better understanding of the complex *Plasmodium* biology as the result of extensive research has led to identification of potential novel targets, including enzymes, transporters, and interacting molecules [53]. For instance, genetic screening of *P. falciparum* identified 2680 genes potentially important in replication and growth, which may represent potential targets [54,55]. However, most triple-therapy combinations and new drugs under development are still in pre-clinical phase (Table 3) or early human trials.

#### 2.3.1. Triple Combination Therapy

Because it is accepted that the development of an ideal single-agent therapy might take years [68], the practical idea of combining three established antimalarial drugs emerged as a solution. In principle, combining more than two medications would prevent the emergence and spread of resistance, since this approach has been successfully implemented against other infectious diseases produced by *Mycobacterium tuberculosis*, *Helicobacter pylori*, and *Human Immunodeficiency Virus* [69]. A phase 2/3, multicenter, open-label, randomized clinical trial was conducted to assess the efficacy and safety of triple artemisinin combination therapies (TACTs) in comparison to ACT for uncomplicated malaria. It was conducted in 18 hospitals in eight countries and compared the efficacy and safety of dihydroartemisinin–piperaquine plus mefloquine and artemether–lumefantrine plus amodiaquine with other ACTs [70]. Both TACTs were well tolerated and efficacious, although vomiting was more frequent with the dihydroartemisinin–piperaquine plus mefloquine combination. QT_C_ interval prolongation (~8 milliseconds) was more common with the combination of amodiaquine to artemether–lumefantrine [70].

The combination of ACT with imatinib (a tyrosine kinase inhibitor used mainly in treatment of leukemias) was also explored in a small phase 2 study [71], based on the rationale that interfering with tyrosine phosphorylation of erythrocyte membrane protein 3 could have an antimalarial effect by preventing merozoite release [72] and potentiating the antimalarial activity of artemisinin in vitro. Furthermore, Chien et al. demonstrated that imatinib combined with dihydroartemisinin–piperaquine produced accelerated parasite clearance that was more pronounced than with dihydroartemisinin–piperaquine alone [71].

#### 2.3.2. Phosphatidylinositol 4-Kinase (PI4K) Inhibitors

The phosphoinositide lipid kinases (PIKs) play an important role in regulating phospholipid biosynthesis, which is essential for *Plasmodium* survival [73]. Targeting phosphoinositide 4 kinase (PI4K) is a particularly attractive therapeutic strategy, because it is required across all *Plasmodium* stages [74]. Two drugs have currently reached clinical trials: KAF156 and MMV390048 [75,76]. KAF156 (phase 2) is an imidazolopiperazine, active against both erythrocytic (asexual and sexual) and pre-erythrocytic liver stages. In a clinical trial conducted in Thailand and Vietnam to assess efficacy, safety, and pharmacokinetics of KAF156 in adults acutely infected with *P. vivax* and *P. falciparum*, single and multiple doses demonstrated an average clearance time of 24 h for *P. vivax* and 45 h for *P. falciparum*. The most common adverse events included sinus bradycardia, thrombocytopenia, hypokalemia, anemia, hyperbilirubinemia, and vomiting [76]. Another phase 1 study aimed to characterize safety, pharmacokinetics, and antimalarial activity of MMV390048, comparing different doses of the drug (40 mg, 80 mg, and 120 mg), and showed greater parasite clearing using 80 mg (clearance half-life of 5.5 h compared to 6.4 h with the 40 mg dose). No serious adverse effects were attributed to the drug itself [75].

#### 2.3.3. *Plasmodium falciparum* P-Type Na^+^ ATPase (*Pf*ATP4) Inhibitors

Sodium homoeostasis is essential for survival of *Plasmodium* in infected RBCs, and the P-type ATPase transporter (*Pf*ATP4) contributes to maintain the necessary low Na^+^ concentration in the intraerythrocytic parasite [77]. Therefore, *Pf*ATP4 inhibitors such as cipargamin (KAE609) and SJ733 are being investigated as antimalarial drugs.

Cipargamin (KAE609) is a spiroindolone analogue, active against all intra-erythrocytic stages of *P. falciparum*, including gametocytes [78,79]. A phase 2, randomized multicenter study conducted in sub-Saharan Africa involving adults with uncomplicated *P. falciparum* malaria demonstrated that single doses (50 to 150 mg) were associated with rapid parasite clearance. Interestingly, clearance was not affected by the K13 mutation associated with artemisinin resistance. However, a mutation in *Pf*ATP4 correlated with 65% of treatment failures [80]. There was concern that cipargamin might cause liver toxicity, but a subsequent randomized controlled trial (RCT) showed no significant difference in liver function tests between cipargamin and artemether–lumefantrine groups [81].

SJ733 ((+)-SJ000557733) is a dihydroisoquinolone currently in phase 2 clinical trials and is the second *Pf*ATP4 inhibitor to enter clinical development [82]. This potent drug is orally bioavailable, rapid-acting, active against all drug resistant *P. falciparum*, and effective against all intra-erythrocytic stages, resulting in transmission blockade [77]. A phase 1a/1b, single-center, open-label, infected-volunteer study was conducted at the University of Tennessee Clinical Research Center showed rapid parasite clearance after a single dose. However, this effect was not sustained, most likely due to rapid drug clearance. The agent was well tolerated with minor side-effects including leukopenia, proteinuria, and bilateral foot paresthesia [83]. Further clinical trials are being conducted to evaluate the combination of SJ733 and CYP3A4 (ClinicalTrials.gov, accessed on 24 November 2022, NCT02661373).

#### 2.3.4. Dihydroorotate Dehydrogenase (DHODH) Inhibitors

Targeting pyrimidine biosynthetic pathways has also been the focus of antimalarial therapeutics exploiting the fact that *Plasmodium* lacks the pyrimidine salvage pathway and blocking the de novo pathway is lethal for the parasite. Dihydroorotate dehydrogenase (DHOHD), an enzyme that catalyzes the rate-limiting step in de novo pyrimidine synthesis (oxidation of dihydroorotate to orotate), is therefore a promising target for drug development [84]. DSM 265 and KAF156 (Ganaplacide) are two potential inhibitors of DHOHD with promising efficacy and safety that are currently in phase 2 human studies [76,85]. A single dose of DSM 265 showed rapid *P. falciparum* clearance but was less impressive against *P. vivax* [85]. KAF156, an imidazolopiperazine active against asexual, sexual, and pre-erythrocytic liver stages of *Plasmodium*, showed effectiveness against *P. falciparum*, including resistant strains with mutations in *P. falciparum* (*Pf*) multidrug resistance (*Pf*MDR), *Pf*Chloroquine Resistance Transporter (*Pf*CRT), and K13 genes which have been impervious to aminoquinoline or artemisinin. Interestingly, none of the patients with recrudescent infection had *Pf* cyclic amine resistance locus (*Pf*CARL) mutations, which are associated with KAF156 resistance [76].

#### 2.3.5. Dihydrofolate Reductase (DHFR) and Thymidylate Synthase (TS) Inhibitors

*Plasmodium falciparum* dihydrofolate reductase (*Pf*dhfr) and thymidylate synthase (TS) is a bifunctional enzyme important in recycling folate and subsequently DNA synthesis. Pyrimethamine and cycloguanil are well-known antifolates used in combating malaria. Unfortunately, the clinical efficacy of those medications has been compromised with the development of *Pf*DHFR mutations at various sites on the enzyme [86]. P218 is a highly selective *Pf*DHFR inhibitor with activity against pyrimethamine-resistant strains, currently in phase 1b clinical trials. A randomized, placebo-controlled volunteer-infection study was conducted to assess for chemoprotection. Two doses of P218 (1000 mg orally 48 h apart) were administered 2 h following parasite inoculation. By Day 28 of inoculation all the patients who received the medication (n = 9) had no evidence of parasitemia, confirming its chemoprotective activity. Side effects were mild and transient, including nasopharyngitis, fatigue, and headache [87].

#### 2.3.6. Isoprenoid Biosynthesis Inhibitors

Isoprenoids, also called terpenoids, are a large family of natural hydrocarbons produced by trees such as oaks, poplars, and eucalyptus. They are required in the asexual replication of *Plasmodium* during post-translational lipid modification of proteins (prenylation) and transfer RNA (tRNA) isopentenylation. In contrast to humans, *P. falciparum* relies exclusively on the 2*C*-methyl-D-erythritol 4-phosphate (MEP) pathway, where the rate-limiting enzyme is *Plasmodium falciparum* 1-deoxy-D-xylulose-5-phosphate reductoisomerase (*Pf*Dxr). Thus, *Pf*Dxr has been the focus of drug therapy development [88].

Fosmidomycin is a phosphonic acid derivative explored as an antibacterial agent against Gram-negative organisms in the late 1970s and 1980s. Because it potently inhibits *Pf*Dxr, multiple studies were carried out to assess its efficacy as a monotherapy or in combination with other artemisinin derivatives. In 2015, a meta-analysis (six pediatric and four adult clinical trials) demonstrated fosmidomycin’s antimalarial efficacy, suggesting the need for additional prospective studies [89].

#### 2.3.7. Choline Transport Inhibitors

Inhibition of phospholipid biosynthesis is another avenue of antimalarial chemotherapy since lipid metabolism is crucial for *Plasmodium* survival. The product of phosphorylation of choline, phosphatidylcholine, is a major lipid component of the parasite’s cell membrane, and various strategies are under investigation to inhibit its production. *Plasmodium* sustains adequate choline supplies through choline transporters, and inhibition of choline transport leads to reduction in phosphatidylcholine biosynthesis and parasite death [90].

Albitiazolium (SAR97276) is a bis-thiazolium-dibromide that acts as a choline analogue blocking the choline transporter and consequently inhibiting phospholipid biosynthesis [91]. It also interferes with heme-detoxification by binding to ferriprotoporphyrin IX [92]. Despite its theoretical potential against *Plasmodium*, both animal and human studies showed lack of efficacy, perhaps due to low oral bioavailability or other unknown mechanisms. More specifically, the unsatisfactory human studies were performed between 2008 and 2012 using intramuscular or intravenous administration, but further development was not pursued [93].

#### 2.3.8. *P. falciparum* Translational Elongation Factor 2 Inhibitors

Protein synthesis is necessary for cell survival in eukaryotic and prokaryotic cells. Inhibiting protein synthesis has been widely utilized as an anti-microbial strategy. For example, doxycycline, clindamycin, and azithromycin have been used against malaria with variable success as they inhibit protein translation in the ribosomes. Inhibition of translation elongation factor 2 (eEF2), which is essential for protein synthesis as it catalyzes GTP-dependent translocation of the ribosome along messenger RNA (mRNA) [94], has been identified as a possible novel target in the treatment of malaria.

M5717 (formerly DDD107498) is a new antimalarial highly selective for *Plasmodium falciparum* translation elongation factor 2 (*Pf*eEF2) with activity against liver, asexual, and sexual stages [95]. The first phase 1, randomized human clinical trial, conducted between 2017 and 2019, showed a long half-life of 146 to 193 h and promising efficacy. The drug was well tolerated, and side effects, including oral hypoesthesia and blurred vision, were noted in some participants at higher doses. However, as with other single-agent therapies, resistance developed after eEF2 mutations arose, highlighting the need to use M5717 in combination [96].

#### 2.3.9. Triaminopyrimidine

ZY-19489 (MMV 253, formerly referred to as compound 12 and AZ-13721412) is a novel anti-malarial drug that belongs to the triaminopyrimidine group. The exact mechanism of action is unknown, and it exhibits potent antimalarial activity towards the asexual blood stage of *P. falciparum* in vitro and in mice models. However, lack of significant activity against liver or sexual forms of the parasite was found. A phase 1, first-in-human healthy volunteer trial showed good efficacy and safety. No resistance was detected in recrudescent cases, suggesting insufficient drug exposure rather than genetic mutation for the suboptimal results. Further studies are necessary to investigate ZY-19489 in combination with a partner drug to cover liver and sexual stages and to prevent recrudescence and resistance [97]. In that regard, Medicines for Malaria Venture (MMV) plans to study ZY19489 in combination with ferroquine, a 4-aminoquinoline that inhibits heme detoxification.

#### 2.3.10. Adhesive Polysaccharide Inhibitors

Malaria can cause severe infection by sequestration of infected erythrocytes, inflammation, and micro-obstruction. Infected erythrocytes attach to the endothelium and other cells using adhesive polysaccharides such as heparan sulfate [98]. Sevuparin (DF02) is a member of the chemical class of heparins. It inhibits merozoite invasion and causes de-sequestration of infected erythrocytes by binding to the N-terminal of the heparan sulfate binding structure of the *Plasmodium falciparum*-infected erythrocyte. It has minimal anticoagulant potency compared to heparin as it does not contain a specific binding sequence for antithrombin. It was shown to be safe and effective when given at doses of 3 mg/kg intravenously 4 times a day for 3 days; this was demonstrated in a phase I/II randomized, open-label, active control study in patients with uncomplicated malaria who were also receiving atovaquone–proguanil [99].

## 3. Conclusions

Despite the development of an extensive pharmacologic armamentarium throughout the years, the burden of malaria remains a global concern contributing significant morbidity and mortality in all age groups. The long-term efficacy of current treatment regimens is threatened by emerging parasite resistance to conventional antimalarial drugs.

However, novel therapeutics are on the horizon to combat malaria. Various innovative drugs are actively in the pipeline for clinical trials to move the field forward. As newer drugs become available, they will be deployed either alone or in combinations extending the viability of existing agents over time in the face of drug resistance. The fast pace of drug development is reassuring overall. However, matching global public health efforts are necessary to address equitable access to emerging life-saving agents for neglected diseases, such as malaria.

## Figures and Tables

**Table 1:** Diagnostic criteria for severe malaria: requires one or more of the following [6].

Species	Signs and Symptoms	Laboratory Findings
*P. falciparum*	Prostration	Acidosis (lactate > 5 mmol/L or plasma bicarbonate < 15 mEq/L or base deficit of >8 mEq/L)
	Convulsions (>2 within 24 h)	Anemia: Hgb < 7 g/dL or hematocrit < 20% in adults (≤5 g/dL or hematocrit ≤ 15% in children < 12 years of age) with a parasite count > 10,000/microliter
	Coma (Glasgow coma scale < 11)	Hypoglycemia < 40 mg/dL
	Abnormal bleeding	Jaundice or bilirubin > 3 mg/dl and parasite count >100,000/microliter
	Circulatory shock	Creatinine > 3 mg/dL or blood urea nitrogen > 56 mg/dL)
	Pulmonary edema, radiologically confirmed or oxygen saturation < 92% on room air with a respiratory rate > 30/minutes	Hyperparasitemia: *P. falciparum* parasitemia > 10%
*P. vivax*, *P. ovale*, and *P. malariae*	Defined as per *P. falciparum* but excluding parasite density thresholds	
*P. knowlesi*	Defined as per *P. falciparum* except parasite density of >100,000/microliter or jaundice and parasite density > 20,000/microliter	

**Table 3:** Potential therapeutic targets and drugs in preclinical stages.

Target	Function	Significance	Drugs in Development	Reference
Malaria Proteases	Degradation of hemoglobin and proteins and aid in cell penetration	Development of the parasite, immune evasion, and activation of inflammation	Not available	[56,57]
Malaria transporters (PSAC, PVM, *Pf*HT, and lactate transporters)	Diffusion of nutrients to infected erythrocyte	Promote growth of the parasite	Preclinical stage (Pentafluoro-3-hydroxy-pent-2-en-1-ones)	[58,59,60,61,62]
V-Type H+ ATPase Channels	Regulate hydrogen ion transportation	Maintain pH for survival of the parasite	Not available	[63]
Aquaporin-3 (AQP3)	Facilitate water and glycerol movement in and out of cells	Parasite survival and replication	Pre-clinical stage (auphen)	[64,65]
Farnesyltransferase	Catalyzing the transfer of farnesyl group from farnesyl pyrophosphate to the C-terminus of proteins containing the CaaX motif	DNA replication, cell division, binding of intracellular proteins to membranes, and protein to protein interaction	Pre-clinical stage (R115777)	[65,66,67]

*Plasmodium* surface anion channel (*P*SAC), parasitophorous vacuolar membrane (PVM), *P. falciparum* hexose transporter (*Pf*HT), and Intravenous (IV).

## Data Availability

Data-sharing not applicable.
